# Differences in Learning Volitional (Manual) and Non-Volitional (Posture) Aspects of a Complex Motor Skill in Young Adult Dyslexic and Skilled Readers

**DOI:** 10.1371/journal.pone.0043488

**Published:** 2012-09-26

**Authors:** Itamar Sela, Avi Karni

**Affiliations:** 1 The Edmond J. Safra Brain Research Center for the Study of Learning Disabilities, University of Haifa, Haifa, Israel; 2 The Segol Department of Neurobiology & Ethology, and the Department of Human Biology, Faculty of Natural Sciences, University of Haifa, Haifa, Israel; University Medical Center Groningen UMCG, Netherlands

## Abstract

The ‘Cerebellar Deficit Theory’ of developmental dyslexia proposes that a subtle developmental cerebellar dysfunction leads to deficits in attaining ‘automatic’ procedures and therefore manifests as subtle motor impairments (e.g., balance control, motor skill learning) in addition to the reading and phonological difficulties. A more recent version of the theory suggests a core deficit in motor skill acquisition. This study was undertaken to compare the time-course and the nature of practice-related changes in volitional (manual) and non-volitional (posture) motor performance in dyslexic and typical readers while learning a new movement sequence. Seventeen dyslexic and 26 skilled young adult readers underwent a three-session training program in which they practiced a novel sequence of manual movements while standing in a quiet stance position. Both groups exhibited robust and well-retained gains in speed, with no loss of accuracy, on the volitional, manual, aspects of the task, with a time-course characteristic of procedural learning. However, the dyslexic readers exhibited a pervasive slowness in the initiation of volitional performance. In addition, while typical readers showed clear and well-retained task-related adaptation of the balance and posture control system, the dyslexic readers had significantly larger sway and variance of sway throughout the three sessions and were less efficient in adapting the posture control system to support the acquisition of the novel movement sequence. These results support the notion of a non-language-related deficit in developmental dyslexia, one related to the recruitment of motor systems for effective task performance rather than to a general motor learning disability.

## Introduction

In a series of studies and theoretical papers during the last two decades, Nicolson and Fawcett developed the ‘Cerebellar Deficit’ theory to explain evidence for non-verbal, sensory-motor, impairments among dyslexic readers [1]–[5]. The theory is an attempt to elaborate on why developmental reading impairment is often accompanied by non-linguistic symptoms; although whether such deficits are a consistent feature of developmental dyslexia has been contested [6]–[10]. The proposal is that the phonological deficits in developmental dyslexia arise from developmental deficits that are not specific to language and that in order to understand developmental dyslexia it is important to identify and consider the neurological substrates of these non-language-related, presumably more basic, deficits. Two specific impairments were suggested: posture and balance deficits ascribed to an unspecified cerebellar dysfunction [1], [11] and deficits in “automaticity” or skill (procedural) learning [12]. However, these deficits have not been verified in several studies[6], [7], [13] and it is not clear whether dyslexic reader individuals suffer from deficits in their posture control system or in their ability to acquire a novel (volitional motor) skill. Here, we tested whether dyslexic readers would show a deficit in the acquisition of a new volitional skill (a manual movement sequence) while maintaining a stable upright posture.

Almost every task (action) related movement of the human body can be conceptualized as consisting of primary (volitional or otherwise) movements, in parallel to posture and equilibrium movements, with the latter providing an effective base for the execution of the primary movements [14], [15]. The acquisition of experience and skill can result in quantitative as well as qualitative changes in task related, primary motor movements [16]–[18]. Postural responses as well can be altered by repeated experience [19]. For example, in adult participants exposed to repeated perturbations, responses become gradually reduced in magnitude and fewer or different muscles were recruited to maintain posture and balance [20]–[22]. It is not clear, however, whether and to what degree the posture and balance control mechanisms in adults can undergo experience-dependent changes when a novel volitional skill is acquired and retained in long-term procedural memory. There are, nevertheless, some indications that posture maintenance movements can be acquired and retained in a time course similar to that of volitional procedural knowledge [23].

The time-course of volitional motor and non-motor skill acquisition has been intensively studied in recent years, and can be conceptualized as a series of distinct phases wherein quantitative but also qualitative changes occur in both performance and the brain representation of the practiced task [16], [17], [24]–[30]. The initial phases of skill learning occur within-session (‘on-line’), starting with a ‘fast learning’ phase in which performance rapidly improves with repeated iterations of the task, but the rate of improvement slows down and subsequently, a plateau phase, in which no additional gains occur despite continued practice is attained [16], [17], [27], [31]. Studies have shown that these within-session gains can be followed by an ‘off-line’, between-sessions, latent phase wherein the gains in performance become resistant to interference and additional performance gains, in both speed and accuracy, may evolve. These later, delayed gains in performance, require time, and often sleep, to be established and expressed [17], [27], [29] reflecting procedural memory consolidation processes [16], [17], [24], [27], [28], [30]. The completion of each phase is essential for a successful initiation of the next phase and for an effective learning process [25], [31].

The purpose of the current study was to test whether young adult dyslexic readers would show a deficit in procedural learning or a dysfunction of the posture and balance control system, or both, when compared to skilled reading peers. To this end, a variant of the hand-writing sequence task developed by Sosnik et al [18], [32] in which both quantitative and qualitative changes in motor performance were described, was designed. In the new task, the Touch Sequence Task (TST) the required movements were performed while standing on a force plate and required the rapid and accurate generation of 3-D hand trajectories. An additional aspect of the TST was that each task-iteration was cued, i.e., was not self initiated, thus affording a measure of pre-motor processing speed. Pre-motor processing time includes aspects such as visual perception [33], [34], decision making [35], the initiation of a movement [31], [36]–[39] as well as the recruitment of motor systems for task performance, engaging a complex feed-forward process and sensorimotor loops [18], [32]. Because participants performed the task while standing on a force plate, several measures of balance and posture maintenance were available for study. The ability to maintain balance and posture can be measured by computing the displacement of the body's center of pressure (COP) on a given surface, in the anterior-posterior and medio-lateral directions. The COP is not a stationary point. Rather, it moves at all times even while standing quietly [40], [41]. The COP location is maintained by: *i*. the ankles which control the posterior/anterior axis of the COP movement through forward or backward corrective motion; and *ii*. movements of the waist that control the medio/lateral axis of the COP movement. Thus, the TST affords the assessment of the training related changes in the execution of the primary, task related, hand movements, the dynamics of balance and posture maintenance, as well as a measure of the experience related changes in readiness and pre-motor processing. Our working hypothesis was that both reading level groups, dyslexic and typical, would demonstrate an ability to acquire the novel sequence of touch movements and show consolidation phase gains as well as effective retention. However, we were expecting, given a recent study on the learning of a finger movement sequence in young female adults with ADD [42] and the data summarized by Nicholson and colleagues on adult dyslexic readers [5], that the memory consolidation phase (expression of between-sessions gains) may be atypical, slower, in the dyslexic readers. We also hypothesized that given that both reading level groups, typical readers as well as the dyslexics, were healthy high-performing young adult university students, both groups would show effective postural adaptation to the task demands on each training-test session, with, perhaps, a slight advantage for the typical readers [1]–[5]. Because the execution of the TST required movements in the medio-lateral axis, stronger adaptation effects were expected in this direction (COPx). Some slowing in reaction time (reflecting pre-motor processing) and in overall task performance was also expected, given previous reports on reduced ‘speed of processing’ in healthy young adult dyslexics [43].

## Materials and Methods

### Participants

Seventeen dyslexic readers (age 28.5±5.1 years, 10 females and 7 males, 14 right handed) and 26 skilled readers (age 24.5±3.4 years, 14 females and 12 male, 22 right handed) were paid to take part in a three-session training program in which a touch sequence task was performed while standing in a quiet stance position. All participants were university students or graduates. Handedness was assessed using the Oldfield questionnaire [44]. The skilled readers were recruited by notices posted on bulletin boards on the university campus. The dyslexic readers were recruited from a large pool of participants through the Student Support Service of the University of Haifa, which assists students with learning disabilities. They were diagnosed as dyslexic during childhood and classified as impaired readers by the Student Support Service using normative reading skills test in Hebrew [45]. The participants in the dyslexic readers' group were only subjects that received a score of −1.5 S.D and below in the MATAL Reading Test (measuring decoding accuracy and fluency in words and pseudowords, reading a text read orally, reading time, and reading comprehension) and who were eligible to receive learning accommodations according the Israeli Higher Education Center for testing and evaluation criteria. No significant between-group differences were found on nonverbal IQ percentile scores, as measured by the Raven Matrices Test [46] and by verbal ability, measured by Similarities [47] (Table 1). All participants were screened with the DSM-IV questionnaire [48] to assess co-morbidity with Attention Deficit/Hyperactivity Disorder (ADHD) (Table 1). Because co-morbidity with ADHD was suggested as a factor in the expression of balance and procedural learning deficits [8], [49], [50] participants with clinically relevant symptoms of ADHD were excluded. Participants reported good health with no history of neurological, medical or musculoskeletal disorders that may affect motor performance, and no chronic use of medications. Informed consent approved by the University of Haifa Ethics Committee was obtained prior to each participant's participation in the study.

**Table 1:** Performance of dyslexic and skilled readers on the IQ tests, the DSM-IV, OMT, background, and reading measurements.

*Test (measure)*	*Dyslexic readers*	*Skilled readers*	*T*	*p*
**Raven**	64.94 (15.59)	65.12 (12.81)	−.14	= .314
**Similarities (SD)**	11.56 (2.26)	11.94 (2.49)	−.898	= .371
**DSM (attention)**	3.65 (2.6)	1.5 (2.2)	2.87	<.01
**DSM (hyperactivity)**	1.06 (1.3)	.38 (.7)	1.9	= .07
**DSM (impulsivity)**	1.24 (1.3)	.46 (.8)	2.2	<.05
**OMT (words correct)**	65.65 (31.2)	112.65 (20.4)	−5.98	<.001
**OMT (words Incorrect)**	2.31 (2.2)	1.81 (2.3)	.7	= .485
**OMT (pseudo-words Correct)**	36.71 (11.6)	62.68 (13.6)	−6.43	<.001
**OMT (pseudo-words Incorrect)**	10.06 (7.8)	5 (4.3)	2.37	<.05
**Fluency (reading time) (s)**	285.49(22.1)	175.53(11.5)	5.99	<.001
**Comprehension**	20.20(3.15)	21.01(1.15)	1.03	= .09
**Phonological accuracy %**	80.5(6.04)	96.11(2.91)	4.98	<.001
**Phonological time (s)**	294.5(85.9)	166.7(35.02)	6.11	<.001
**Orthographic correct %**	88.21(8.0)	98.4 (4.1)	5.78	<.001
**RAS time (s)**	32.64 (5.61)	27.25 (5.41)	4.36	<.001
**RAN (s)**	35.31 (8.41)	26.42 (3.21)	5.68	<.001
**Digit Span (forward and backward computed Standard score)**	10.45 (7.18)	12.91(3.24)	4.59	<.001

### Background and reading measurements

#### General ability

Performance ability was measured by the Raven Matrices Test [46], in which the participants were introduced to visual matrices with one part of the matrix missing, and had to choose, from four options, the one that matched the missing part, based on missing patterns and orientation. Verbal ability was measured by Similarities subtest WAIS 2003 [47].

### Reading Measurements

1. Decoding- one list of 168 words and one list of 96 pseudowords [51] were given to the participants. The Participants were asked to read each list in a minute and the number of correct items read in each of the lists was documented.
2. Fluency: Oral reading task [51]. In this task, the participants were asked to read a text (276 words) and the reading time was measured.
3. Comprehension: Silent reading comprehension test [52] containing 319 words and 24 multiple choice questions.

### Word Recognition

1. Phonological Processing [51] contained 1. Phonemic Deletion (total score: 25) and b. Segmentation Test (total score: 16). The accuracy (the number of correct answers) and the total response time were measured for the two tests and combined in one score.
2. Orthographic Processing: Parsing Test [53] containing 46 rows of words. Each line contained four words printed without spaces between them. The participants had to identify the words in each row by drawing a line to specify where the spaces should be. Performance accuracy was measured.

### Cognitive abilities

1. RAS Naming [54].
2. RAN Naming letters [54].
3. Short term memory and capacity- digit span backward and forward subtests standard score WAIS-III [47].

### Experimental Measures

#### Apparatus

**Figure 1:**
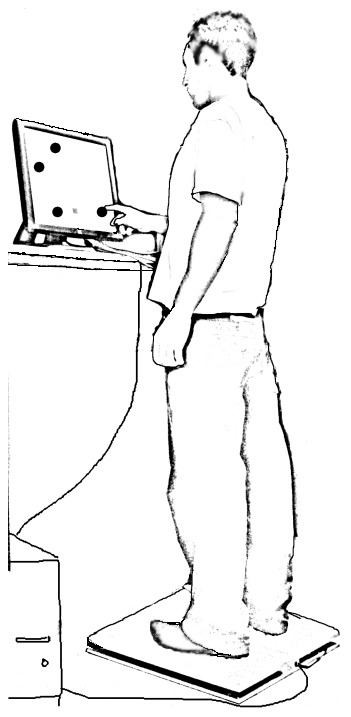
The experiment setup. The participant was instructed to stand on the forceplate in a quiet stance position, in front of the touch screen. The forceplate's output data was streamed into the same desktop PC that presented and controlled the touch sequence task.

The training-test setup included a touch-sensitive (19″ ELO Touchsystems) screen and an AMTI AccuSway balance and posture sway measurement forceplate (Advanced Mechanical Technology, Inc., Watertown, MA). The touch screen, which was used to display the touch sequence targets and to collect touch times, was positioned in front of the forceplate at a distance of approximately 50 cm from the participant's shoulder at a height of approximately 170 cm (Figure 1). The distance to the touch screen was set for each individual separately to ensure slight flexion of the elbow during performance of the task. The touch sequence presentation and response recordings as well as the forceplate output recording were controlled by a desktop computer using an in-house application and the AMTI data collection software. Matlab software (Version 2008a, The MathWorks, Natick, MA) was used to prepare data for statistical analysis with SPSS (Version 15, SPSS Inc., Chicago IL).

#### The Touch Sequence Task (TST)

**Figure 2:**
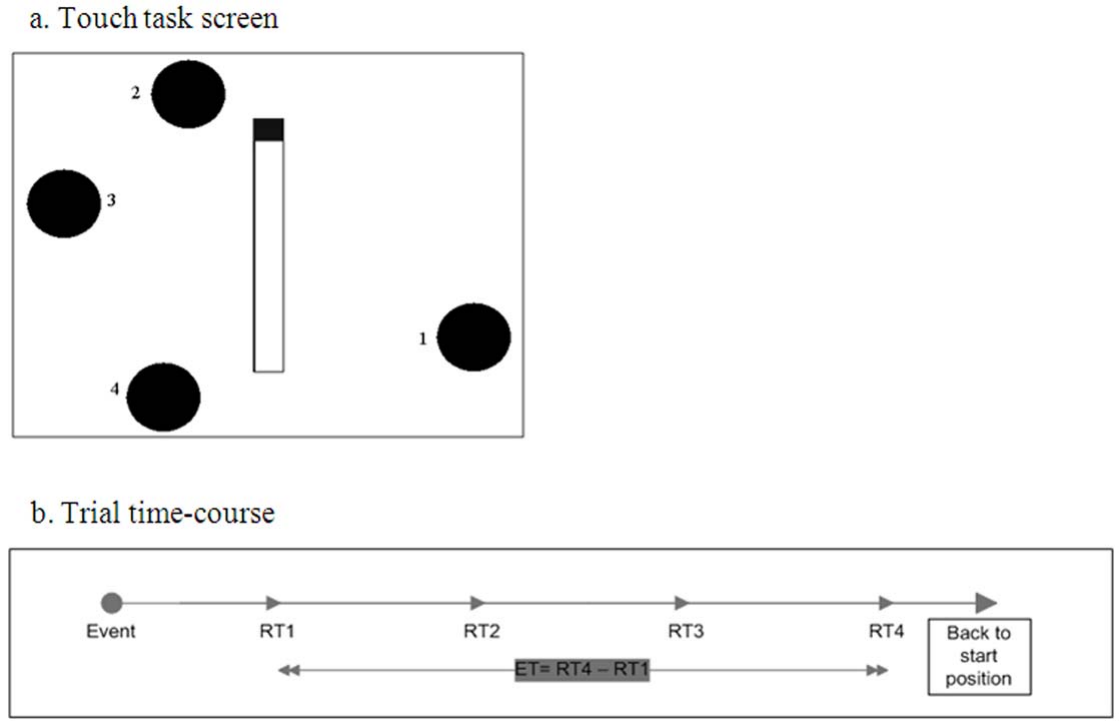
Task screen and time-course of a single trial. a) The relative positions of the four targets (black circles) (the numbering is for illustration purposes only and was not included in the actual display). As the targets changed color from black to yellow, the participant was instructed to turn them back to black by touching them, as fast as possible, following a predefined order (1→2→3→4), and to return to the starting point and wait for the next trial to begin. b) The time-course of a given trial was divided into two continuous time periods: RT1 (Reaction Time for target 1)- the time between the ‘start’ cue (the targets' color change) and the touching of the first target. ET (Execution Time) - the time between touching the first target and touching the fourth target.

Participants were trained to perform a sequence of touch movements on the touch-sensitive screen using the index finger of the dominant hand with minimal body movements. The touch targets were four black circles (diameter 45 mm) that appeared on the screen in the same four locations (Figure 2a) throughout the three sessions of training. The touch targets were arranged in a left-right flipped manner for left-handed individuals.

The participants were instructed to stand in a quiet stance position with the index finger of their dominant hand located near, but not touching, the first target (the lower right target for right-handed participants and the lower left for left-handed individuals), and to wait for the cue to begin the trial. The start cue was a simultaneous change of all four target circles from black to yellow. The participants were instructed to touch the targets, as quickly and as accurately as possible, in the given sequence (Figure 2a). The targets were to be touched only in the predefined order in a counter-clockwise direction (clockwise for left-handed individuals). A switch in color from yellow back to black signaled a correct touch (on target and in the right order). Participants were instructed to return to the starting position with the index finger just above, but not touching, the first target circle after completing each touch sequence and wait for the next trial to start. There was a set interval of three seconds between the beginning of each trial and the following trial.

A successful trial was defined as one in which the participant did not miss any of the 4 targets in the specified sequence. Visual indications of current trial performance and the individual's performance relative to his or her best performance were provided in the form of a small time indicator that went on when the touch sequence was initiated and was stopped when the final target was touched. A small arrow indicated the best time achieved within the current block.

#### Procedure

Each participant took part in three identical sessions: initial training (first session), 24 hours later (second session), and one week after the initial training session (third session). Task instructions were provided at the beginning of each session and participants were allowed to touch the screen in multiple random sequences to familiarize themselves with its activation. Feedback for an effective touch was provided by the movement of the mouse cursor to the touched location. The training-testing blocks were initiated only when the participant and the experimenter were both satisfied with the participant's ability to consistently produce effective touch movements. Each training-testing block consisted of a 5-second interval of quiet stance followed by 20 touch sequence trials (60 seconds) and a final 5-second interval of quiet stance. Ten blocks were performed in each session.

#### TST performance analysis

Accuracy and speed were calculated for each block. The measure for accuracy was the percentage of trials within the block in which at least one target circle was missed (the error rate, ER). The measure for speed of performance was the average time for completing the sequence in each block of trials. Unsuccessful trials and successful trials that immediately followed unsuccessful ones were excluded from the analysis. This ‘double exclusion criteria’ allowed for the included trials to be more unified.

The time for completing the sequence in each trial was divided into two sub-periods, intervals, which were analyzed separately (Figure 2b). The first sub-period was the time from the trial start cue (the targets' color change) to the time when the first target was touched (RT1). This interval was considered to reflect task initiation, including visual perception and the decision to respond to the ‘go’ cue, as well as the time for preparation and execution of the first touch movement. The second interval included the touch times and movement times starting with the first target touch and ending with the fourth target touch (sequence execution time, ET). ET was calculated by subtracting the touch time of target 1 from the touch time of target 4 (ET** = **RT4-RT1).

#### Non-volitional task performance COP Analysis

**Figure 3:**
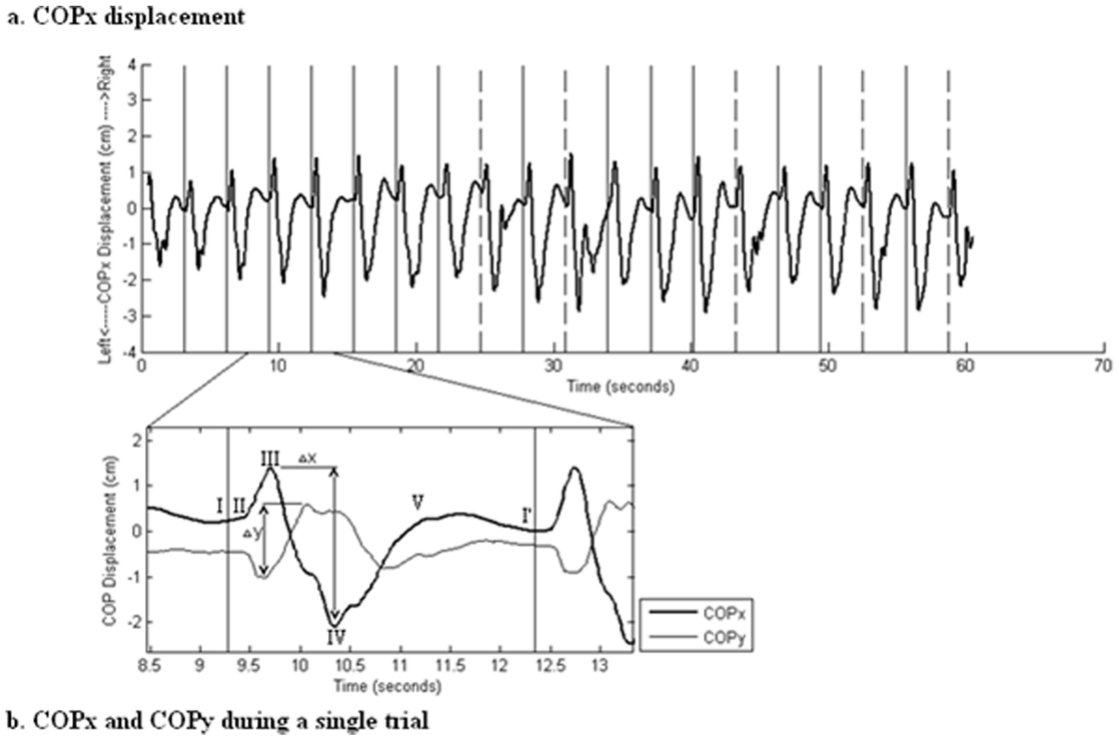
COP displacement during a block and a single trial. a) Representative results of COPx displacement during a block. Vertical solid lines represent the ‘start’ event of a successful trial. Vertical dashed lines represent the ‘start’ event of an unsuccessful trial. b) Detailed examination of the COPx (medio-lateral) and COPy (anterior-posterior) sway movements displacements during a trial (positive values are right and backward displacement of the COPx and COPy, respectively): (I) the ‘start’ cue, (II) as the hand moves toward the first target the COP starts to travel to the right and toward the screen, (III) as the hand starts to move toward target 2 the COP stops traveling to the right and forward and begins its shift backward and left, (IV) as the hand touches targets 2 and 3 and moves toward target 4, the COP stops traveling backward and left and turns again to forward and right, (V) the trial ends as the hand returns to its original location and the body posture is stabilized; (I′) the beginning of the next trial. Δx and Δy are the absolute difference between the extreme values in each of the COP axes within a given trial; SDΔx and SDΔy are the standard deviations of all successful trials Δx and Δy within a block, respectively.

The maximal amplitude of the movement of the center of pressure (COP) in the x (Δx) and y (Δy) directions (medio-lateral and anterior-posterior sway movements, respectively) within each trial, as well as the trial by trial block variance (standard deviations) of the maximal amplitude of the movement of the COP in both directions (SDΔx, SDΔy) were computed as measures of posture maintenance. Representative results of COPx and COPy displacements during a block of touch-sequence task iterations are shown in Figure 3. The COP is the point location of the vertical ground reaction force vector, representing a weighted average of the pressure vectors on the contact surface [40], [55]. Block by block and session by session changes in these parameters were considered as measures of experience-dependent changes in posture control. It was hypothesized that as the main axis that is used in the current study's task is the medio-lateral axis, stronger learning effects will be found in the COPx as compared to the COPy.

## Results

### Background and reading measurements

T-test analyses revealed significant differences between the two reading groups on decoding reading fluency word identification and cognitive measures. The dyslexics read significantly fewer words and pseudowords in a minute and were slower in text reading than the controls. They also obtained lower scores in the phonological and orthographic tests as well as in the RAS and RAN naming tests. However no significant differences were found between the two groups in general ability (Raven and Similarity Tests) attention (DSM) and in reading comprehension test (Table 1).

### Experimental measurements

**Figure 4:**
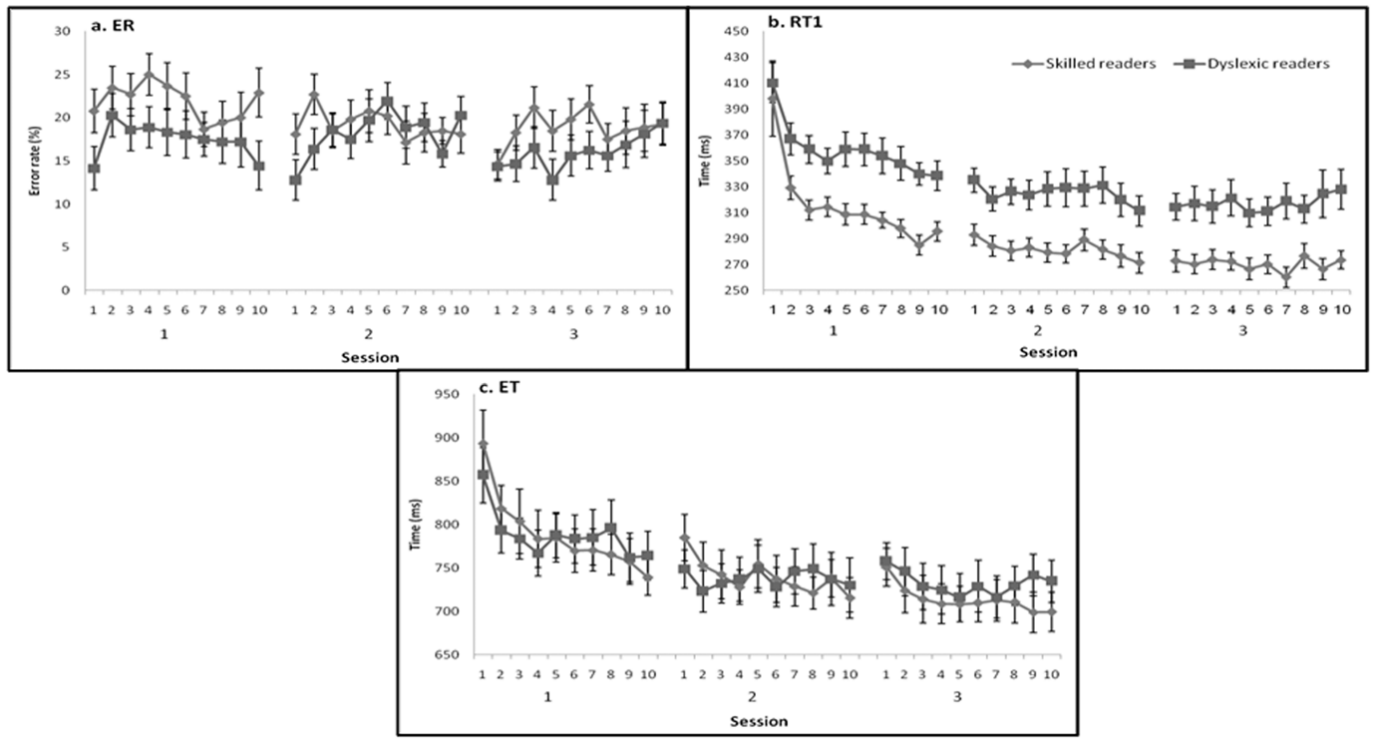
The group mean value of the volitional manual task parameters for dyslexic and skilled readers throughout the training program. a. ER. b. RT1, and c. ET. Error bars indicate group standard error.

**Figure 5:**
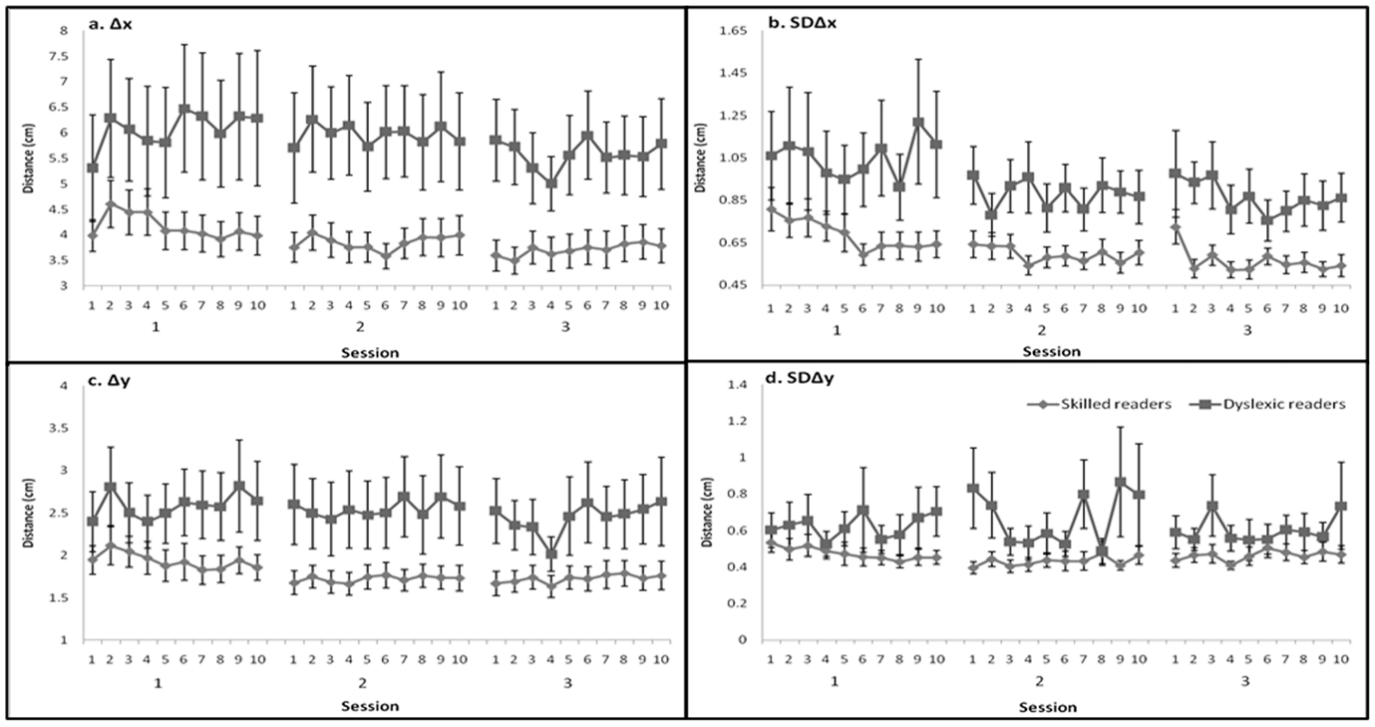
The posture control parameters throughout the training program in the dyslexic and skilled readers. a. Δx – block mean value of the maximal amplitude of the movement of the COP in the medio-lateral direction within a given trial; b. SDΔx – block standard deviation of the individual trials' Δx. c. Δy – block mean value of the maximal amplitude of the movement of the COP in the anterior-posterior direction within a given trial; d. SDΔy – block standard deviation of the trials' Δy. Error bars indicate group standard error.

Training on the given set of hand movements led to significant within- and between-session improvements in both the volitional touch-sequence task performance (Figure 4) and the non-volitional, posture control measures (Figure 5), but these measures were differentially affected by training. The training led to significant gains in performance by both reading-level groups. Nevertheless, the dyslexic readers showed smaller learning effects than the skilled readers and in some parameters failed to show significant experience-dependent changes.

The results of the analyses of variance (ANOVAs) for the TST performance measures (ER, RT1, ET) and the postural movements' parameters (Δx, Δy, SDΔx, SDΔy) are summarized in Tables 2 and 3, respectively. Each parameter was analyzed separately with the two reading-level groups as the between-subject factor and with the three sessions and the 10 blocks comprising each session as within-subject factors. In addition, to test for within-session gains the first and final 4 blocks of the sessions were compared. To test for between-session gains, the final 4 blocks of a session were compared to the initial blocks of the subsequent session. Thus, time-point (2) and blocks (4) were within-session factors. Whenever sphericity was not supported, the Greenhouse-Geisser method was used to correct the degrees of freedom and determine the significance of results.

**Table 2:** ANOVA results for the volitional manual movement parameters for a) skilled and b) dyslexic readers.

*Group*	*Comparison*	*ER*	*RT1*	*ET*
a) Skilled readers	Sessions effect	F_(2, 50)_ = 4.99**	F_(2, 50)_ = 41.16***	F_(2, 50)_ = 26.55***
	Blocks effect	F_(2, 50)_ = 2.39*	F_(9, 225)_ = 11.18***	F_(9, 225)_ = 15.41***
	Sessions and blocks interaction	F_(2, 50)_ = .64 (NS)	F_(18, 450)_ = 6.54***	F_(18, 450)_ = 2.63***
	1^st^ session within-session gains	F_(1, 25)_ = 2.18 (NS)	F_(1, 25)_ = 24.64***	F_(1, 25)_ = 15.92***
	2^nd^ session within-session gains	F_(1, 25)_ = 2.34 (NS)	F_(1, 25)_ = 1.03 (NS)	F_(1, 25)_ = 15.61**
	3^rd^ session within-session gains	F_(1, 25)_ = .16 (NS)	F_(1, 25)_ = .55 (NS)	F_(1, 25)_ = 4.23*
	2^nd^ to 1^st^ between-session gains	F_(1, 25)_ = 12.32**	F_(1, 25)_ = 38.06***	F_(1, 25)_ = 23.69***
	3^rd^ to 2^nd^ between-session gains	F_(1, 25)_ = .00 (NS)	F_(1, 25)_ = 13.40***	F_(1, 25)_ = 10.95**
	2^nd^ session delayed gains	F_(1, 25)_ = .96 (NS)	F_(1, 25)_ = 8.59**	F_(1, 25)_ = .50 (NS)
	3^rd^ session delayed gains	F_(1, 25)_ = .12 (NS)	F_(1, 25)_ = 7.39**	F_(1, 25)_ = .02 (NS)
b) Dyslexic readers	Sessions effect	F_(1.37, 23.33)_ = .57 (NS)	F_(1.27, 20.34)_ = 20.69***	F_(2, 32)_ = 5.99**
	Blocks effect	F_(9, 153)_ = 1.51 (NS)	F_(3.73, 59.70)_ = 2.87**	F_(3.01, 48.15)_ = 3.44*
	Sessions and blocks interaction	F_(18, 306)_ = 1.12 (NS)	F_(6.25, 100.07)_ = 4.60***	F_(18, 288)_ = 2.38**
	1^st^ session within session gains	F_(1, 16)_ = .781 (NS)	F_(1, 16)_ = 11.84**	F_(1, 16)_ = 2.01(NS)
	2^nd^ session within session gains	F_(1, 16)_ = 2.96 (NS)	F_(1, 16)_ = .29 (NS)	F_(1, 16)_ = .43 (NS)
	3^rd^ session within session gains	F_(1, 16)_ = 2.80 (NS)	F_(1, 16)_ = .37 (NS)	F_(1, 16)_ = .58 (NS)
	2^nd^ to 1^st^ between sessions gains	F_(1, 16)_ = .26 (NS)	F_(1, 16)_ = 24.26***	F_(1, 16)_ = 1.24***
	3^rd^ to 2^nd^ between sessions gains	F_(1, 16)_ = 1.77 (NS)	F_(1, 16)_ = 3.65 (NS)	F_(1, 16)_ = .12 (NS)
	2^nd^ session delayed gains	F_(1, 16)_ = .14 (NS)	F_(1, 16)_ = 17.99***	F_(1, 16)_ = 15.20***
	3^rd^ session delayed gains	F_(1, 16)_ = 7.70*	F_(1, 16)_ = 1.03 (NS)	F_(1, 16)_ = .63 (NS)

The session and block effects, interaction between sessions and blocks, as well as the within-session, between-session, and delayed gains effects, were analyzed using separate ANOVAs on ER, RT1, ET.

* p<.05,

** p<.01,

*** p<.001; NS– not significant.

**Table 3:** ANOVA results for the posture maintenance parameters for a) the skilled readers and b) the dyslexic readers.

	*Comparison*	*Δx*	*SDΔx*	*Δy*	SDΔy
a) Skilled readers	Sessions effect	F_(2, 50)_ = 2.25 (NS)	F_(1.52, 38.11)_ = 5.70**	F_(1.46, 36.69)_ = 3.11*	F_(1.55, 38.76)_ = 1.76 (NS)
	Blocks effect	F_(9, 225)_ = .82 (NS)	F_(5.53, 7.29)_ = 3.80***	F_(3.86, 96.62)_ = .62 (NS)	F_(5.01, 125.37)_ = .20 (NS)
	Sessions and blocks interaction	F_(18, 450)_ = 2.02 (NS)	F_(8.85, 221.32)_ = 1.18 (NS)	F_(4.58, 114)_ = 1.19 (NS)	F_(7.27, 181.88)_ = 1.01 (NS)
	1^st^ session within-session gains	F_(1, 25)_ = 2.33 (NS)	F_(1, 25)_ = 8.88**	F_(1, 25)_ = 2.42 (NS)	F_(1, 25)_ = 4.45*
	2^nd^ session within-session gains	F_(1, 25)_ = .18 (NS)	F_(1, 25)_ = 1.08 (NS)	F_(1, 25)_ = .28 (NS)	F_(1, 25)_ = 1.49(NS)
	3^rd^ session within-session gains	F_(1, 25)_ = 1.23 (NS)	F_(1, 25)_ = 2.20 (NS)	F_(1, 25)_ = 1.79 (NS)	F_(1, 25)_ = 1.36 (NS)
	2^nd^ to 1^st^ between-session gains	F_(1, 25)_ = 1.88 (NS)	F_(1, 25)_ = 6.35**	F_(1, 25)_ = 4.02*	F_(1, 25)_ = 3.95 (NS)
	3^rd^ to 2^nd^ between-session gains	F_(1, 25)_ = 1.12 (NS)	F_(1, 25)_ = 1.12 (NS)	F_(1, 25)_ = .00 (NS)	F_(1, 25)_ = 2.78 (NS)
	2^nd^ session delayed gains	F_(1, 25)_ = .42 (NS)	F_(1, 25)_ = .34 (NS)	F_(1, 25)_ = 3.57 (NS)	F_(1, 25)_ = 2.12 (NS)
	3^rd^ session delayed gains	F_(1, 25)_ = 2.63 (NS)	F_(1, 25)_ = .06 (NS)	F_(1, 25)_ = .45 (NS)	F_(1, 25)_ = .00 (NS)
b) Dyslexic readers	Sessions effect	F_(1.24, 19.82)_ = .24 (NS)	F_(1.20, 19.30)_ = 1.45 (NS)	F_(1.41, 22.63)_ = .17 (NS)	F_(1.27, 20.47)_ = .67 (NS)
	Blocks effect	F_(2.07, 33.19)_ = .74 (NS)	F_(2.96, 47.44)_ = .81(NS)	F_(2.47, 39.4)_ = 1.18 (NS)	F_(1.59, 25.44)_ = 1.25 (NS)
	Sessions and blocks interaction	F_(18, 288)_ = .98 (NS)	F_(18, 288)_ = .80 (NS)	F_(18, 288)_ = .10 (NS)	F_(18, 288)_ = 1.26 (NS)
	1^st^ session within-session gains	F_(1, 16)_ = .77 (NS)	F_(1, 16)_ = .15 (NS)	F_(1, 16)_ = .35 (NS)	F_(1, 16)_ = .18 (NS)
	2^nd^ session within-session gains	F_(1, 16)_ = .06 (NS)	F_(1, 16)_ = .42 (NS)	F_(1, 16)_ = .92 (NS)	F_(1, 16)_ = 1.00 (NS)
	3^rd^ session within-session gains	F_(1, 16)_ = .18 (NS)	F_(1, 16)_ = 1.56 (NS)	F_(1,16)_ = 1.89 (NS)	F_(1, 16)_ = .23 (NS)
	2^nd^ to 1^st^ between-sessions gains	F_(1, 16)_ = .08 (NS)	F_(1, 16)_ = 1.96 (NS)	F_(1, 16)_ = .02 (NS)	F_(1, 16)_ = .98 (NS)
	3^rd^ to 2^nd^ between-sessions gains	F_(1, 16)_ = .34 (NS)	F_(1, 16)_ = .01 (NS)	F_(1, 16)_ = .48 (NS)	F_(1, 16)_ = .74 (NS)
	2^nd^ session delayed gains	F_(1, 16)_ = .66 (NS)	F_(1, 16)_ = 3.11 (NS)	F_(1, 16)_ = .51 (NS)	F_(1, 16)_ = .67 (NS)
	3^rd^ session delayed gains	F_(1, 16)_ = .46 (NS)	F_(1, 16)_ = .52 (NS)	F_(1, 16)_ = 1.62 (NS)	F_(1, 16)_ = .96(NS)

The session and block effects, interaction between sessions and blocks, as well as the within-session, between-session, and delayed gains effects were analyzed using separate ANOVAs on Δx, SDΔx, Δy, and SDΔy.

* p<.05,

** p<.01,

*** p<.001; NS– not significant.

### Manual (TST) performance

#### ER

The skilled readers showed an overall significant main effect for both sessions and blocks (Table 2a), indicating a significant improvement in the accuracy of task performance throughout the three sessions (Figure 4a). There was no significant interaction between sessions and blocks, indicating a similar rate of improvement across blocks within the three sessions. No significant gains in accuracy were found within any of the three sessions (Δ% = −2.75; −1.77; .38, for the first, second and third sessions, respectively) although there was a significant difference decrease in the average ER across the three sessions (ER = 21.9%; 19.2%; 18.79%, for the first, second and third sessions, respectively).

The dyslexic readers group showed no significant main effects for either sessions or blocks (Table 2b), indicating no significant reduction in the number of error trials during training (Figure 4a). There were no significant gains either within the sessions or between the sessions, although significant delayed gains occurred between the end of the second session and the beginning of the third.

There was no significant group effect when the dyslexic readers were compared to the skilled readers (F_(1, 41)_ = 1.08, p = .18, Figure 4a), and no significant group by sessions or group by blocks interactions (F_(2, 82)_ = 1.46, p = .18; F_(9, 369)_ = .98, p = .66, respectively). The dyslexic readers committed fewer errors during the first session compared to the skilled readers (17.4% vs. 21.9%) but the difference was not significant (F_(1, 41)_ = 2.74, p = .10). The between groups gap in error rates decreased to less than 2% at the second and third sessions.

#### RT1

The skilled readers showed a significant main effect for both sessions and blocks (Table 2a), with increasingly shorter RT1 intervals across the sessions and within the sessions across blocks (Figure 4b). However, there was a significant interaction of sessions and blocks, reflecting the fast improvement within the first session (a mean difference of 43 ms between the initial four and final four blocks of the session) but no significant within-session improvements in sessions 2 and 3 (a mean difference of 5.6 ms and 3 ms, respectively). There was significant fast learning during the initial blocks of the first session indicating a novelty effect [17], but by the end of the first session performance was almost at asymptote. There was a significant difference in RT1 between sessions, and significant gains in performance that evolved between-sessions (i.e., significant delayed gains) (Figure 4b). Thus, RT1 was significantly reduced at 24 hours after the termination of the first training session. Also, there was a small but significant improvement across the week-long interval between sessions 2 and 3.

The dyslexic readers showed significant training effects for both sessions and blocks (Table 2b) as well, with a significant improvement in speed throughout the training program (Figure 4b). There was also a significant sessions by blocks interaction, with significant within-session gains only in the first session. There were clear between-sessions gains: mean RT1 values of 358 ms, 325 ms and 317 ms in the first, second, and third sessions, respectively, with significant delayed gains after the first, but not the second, session.

A comparison of the RT1 of dyslexic readers and skilled readers yielded a significant group effect (F_(1, 41)_ = 14.19, p = .001). Nevertheless, both groups showed effective gains in RT1 (Figure 4b) and there was no significant group by sessions interaction (F_(2, 82)_ = .20, p = .82), and no group by blocks interaction (F_(9, 378)_ = 1.27, p = .38). Although both groups started at a nearly equal level of performance (approximately 400 ms in the first block of the first session) (Figure 4b), a significant time-gap (40–50 ms) developed by the second block of the first session in favor of the skilled readers. This time gap was maintained throughout the training program; thus, RT1 by the end of the third session was 273 ms in the skilled readers and 327 ms in the dyslexic readers.

#### ET

Similar to RT1, the skilled readers significantly improved the sequence ET throughout training with a significant main effect of both sessions and blocks (Figure 4c). A significant interaction effect was found between sessions and blocks: ET significantly diminished throughout and within all three sessions, but at a different rate across sessions. Significant between-session effects were also found (Table 2a).

The dyslexic readers group also exhibited significant sessions and blocks effects (Table 2b), with a significant reduction in ET throughout the training period, and a sessions and blocks interaction. There was a significant improvement between the first and second sessions; however, there were no significant within-sessions gains in any of the three sessions. There were significant delayed gains only after the end of the first session.

There was no significant group effect when the two groups of readers were compared (F_(1, 41)_ = .00, p = .99) and no significant group by sessions (F_(2, 82)_ = .59, p = .59) or group by blocks (F_(3.76, 154.32)_ = 2.31, p = .06) interaction. Both groups demonstrated a robust, significant reduction in ET throughout the training program (Figure 4c).

### Posture control

There were significant changes in posture, reflected in the COP measures, as participants executed the touch sequence task. As can be seen in Figure 3b, participants spent the between-trial intervals (on average about 2 seconds) in a quiet stance position and the COP remained relatively stable. When a trial was initiated, the COP shifted from its original location toward the first target to the right (COPx). Then, as the performing hand moved to targets 2 and 3, the COP was shifted to the left. Finally, to facilitate the touching of the fourth target and the return to the starting position, the COP shifted to the right (with a small overshoot). In the orthogonal axis (COPy, Figure 3b), the initial shift of the COP was oriented forward, toward the touch screen. As the trial progressed and the hand moved toward the second, third, and fourth targets, the COP shifted backward with an overshoot; it was re-stabilized in close proximity to the starting location, while participants were waiting for the next trial to start (Figure 3b).

The working hypothesis was that the establishment of a posture control routine would be manifested by decreasing values for SDΔx and SDΔy rather than for Δx or Δy [56]. Also, because movement in the medio-lateral axis reflected more closely the required volitional movement sequence, changes in this axis (Δx and SDΔx) were expected to be more task specific than in the orthogonal antero-posterior axis (Δy and SDΔy).

#### Δx and SDΔx

The skilled readers (Table 3a) showed no significant changes in Δx (Figure 5a) as a function of training, either within or between the training sessions. There was, however, a robust decrease in variance (SDΔx) across the three sessions (comparing the initial 4 blocks of the first session to the final 4 blocks of the third session) in 22 of the 26 skilled readers, with each individual experiencing, on average, 45% less variance in Δx across the task iterations within the test block. Altogether, SDΔx significantly decreased across blocks within the training sessions (Table 3a), as well as between the first and second sessions (Figure 5b). The average value of SDΔx tended to increase at the beginning of the third session, but this trend was reversed in the second block, with a significant decrease in the mean value of SDΔx in the following 9 blocks (t_(25)_ = 2.98, p<.01).

#### Δy and SDΔy

The skilled readers also showed a significant decrease in the value of Δy across sessions (Table 3a, Figure 5c). There was a significant decrease in Δy between the first and second sessions but not between the second and third sessions. Similarly, except for a rapid decrease in SDΔy within the first session (Figure 5d), there was no indication of an experience-dependent change in terms of COP movements in the y direction.

In contrast to the robust learning effects in the manual aspect of the touch sequence task, and the training effects in the skilled readers' posture-related parameters, the dyslexic readers showed no training effects in any of the 4 parameters that reflected the posture control system (Table 3b). There were significant group effects for parameters Δx (Figure 5a), SDΔx (Figure 5b), and Δy (Figure 5c) (F_(1, 41)_ = 7.15, p = .01, F_(1, 41)_ = 7.93, p<.01, F_(1, 41)_ = 5.33, p<.05, respectively), but not for SDΔy (Figure 5d) (F_(1, 42)_ = 3.65, p = .06). There were no significant group-by-sessions interactions in any of the posture control system parameters (F_(1.28, 52.65)_ = .05, p = .95; F_(1.26, 51.67)_ = .37, p = .59; F_(1.43, 58.84)_ = .29, p = .67; F_(1.67, 68.75)_ = 1.92, p = .16, for Δx, SDΔx, Δy, and SDΔy, respectively).

### The relationships between attention, motor learning and dyslexia

Pearson correlation analyses were applied to test for a possible relationship between the TST performance measures (the values of ER, RT1, ET) and the SDΔx in each of the three training sessions, and the DSM Attention and ADHD scores (as assessed in the screening phase). Among the skilled readers, significant correlations were found between ET in each of the three sessions and the Attention score (r = .529, p<.01; r = .618, p = .01; r = .669, p<.001; for the first, second, and third sessions, respectively). No significant correlations were found between the skilled readers' posture control and the DSM variables. No significant correlations were found between any of the above variables among the dyslexic readers.

## Discussion

Altogether, the skilled readers' results support the notion that the balance control system is recruited to support the acquisition of a new volitional motor movement sequence. Moreover, the current results suggest that although the dyslexic readers were as effective as skilled readers in their abilities to acquire and robustly retain the trained movement sequence, they were impaired in the recruitment and integration of some aspects of the task compared to skilled readers. The current results therefore, support the notion of a non-language-related deficit in developmental dyslexia [57], one related to the recruitment of motor systems for effective task performance. Nevertheless, the results do not support a strong notion [3] of a general procedural (motor) learning disability as a core deficit in developmental dyslexia.

### Learning the volitional aspects of the task

The skilled readers showed robust improvement in the performance of the explicitly trained volitional manual task. The time-course of learning as indicated by the three performance measures (RT1, ET, and ER), reflected distinctive phases; phases that were suggested as characteristic of procedural skill acquisition, specifically motor sequence learning [16], [17], [31]. However, the current results suggest that different aspects of the task may require a different amount of practice to be mastered. Fast within-session gains were noted in the first training session on all three parameters; however, within-session gains in ET occurred also during the second and even the third sessions. Clear delayed, ‘off-line’ gains [17], [30], [58] were apparent only for RT1. These differences suggest that different aspects of the task may require different amounts of experience (practice) to be mastered. That is, different amounts of practice may be needed to attain sufficient experience, to continue to a subsequent stage in the learning process, for different task aspects [25], [31]. Previous studies have shown that a minimum of task iterations is needed in order to successfully shift between a given phase in the learning process to the next; for example, the triggering of delayed gains may require the attainment of asymptotic performance (plateau phase) within the session [17], [25], [31], [59]. The delayed gains in RT1, expressed in sessions 2 and 3, can be accounted for by the plateau phase attained at the end of the first and second sessions, respectively. On the other hand, the length of each training session was apparently insufficient for triggering robust delayed gains for ET. ET continued to improve throughout each of the consecutive training sessions (a significant within-session learning effect was noted in all three sessions), and in terms of this parameter, task performance failed to reach any level of saturation in any of the three sessions. Thus, it may be that the fast learning phase was not successfully completed in any of the training sessions so the next phase in the learning process could not be initiated [17], [25], [31], [59].

This conjecture suggests that different task parameters reflect different aspects and perhaps sub-systems engaged in the performance of the task. Thus, RT1 may encompass processes such as visual perception [33], [34], decision making [35], and motor planning, as well as the initiation of the motor movement sequence [31], [36]–[39]. ET may reflect different processes involved in the appropriate choice, ordering, and timing of the sequence of the different pointing movements that constitute the task, as well as processes such as hand-eye coordination needed to maintain accuracy [18], [32]. Nevertheless, the same parameter may reflect different sub-systems and sub-routines at different levels of experience (stages of practice), a notion proposed to explain behavioral and brain imaging data indicating that the performance of a given task may be sub-served by different neural representations at different levels of experience [16], [17], [60], [61]. In later stages of practice, for example, the movement routine employed for task performance may change through processes such as chunking or co-articulation [18], [60], [62], which presumably may be differentially reflected in changes in RT1 and ET [37]. From this perspective, a number of sub-systems (i.e., neural representations) may have been subject to experience-driven modification across the three training sessions, and the improvement of different performance measures was expressed at different rates (Table 2a).

The current results clearly show that the dyslexic readers were very effective in learning the volitional (manual) aspects of the movement sequence, in retaining the gains between sessions (intervals of a day and a week) and even in improving upon their performance in subsequent sessions. There were robust gains and no significant group differences in the speed of sequence execution (ET). The dyslexic readers were able to execute the sequence of movements as quickly as the skilled readers and showed significant and robust improvement in movement speed as a function of experience and time. This suggests that there was no deficit in their ability to execute and to learn from experience a given sequence of volitional motor movements. These gains in speed were not at the expense of accuracy in both reading-level groups. Indeed, the dyslexics tended to be more accurate in their performance. Thus, in both reading-level groups the gains in speed cannot be explained as reflecting a speed-accuracy tradeoff [16], [17], [63].

RT1 was the only measure in which a significant group difference was found. Nevertheless, both groups showed effective gains in RT1 (Figure 4b) and there were no significant group differences in the rate of improvement. The significant difference between the groups, expressed in RT1, reflected the opening of a time-gap (40–50 ms) early on in the first session in favor of the skilled readers. This time-gap in RT1 was maintained throughout the experiment. The finding that the dyslexic readers' improvements in RT1 paralleled those of the skilled readers (Figure 4b), suggests that the ability of the dyslexic readers to improve on the initiation of the sequence, as a function of accumulating experience, did not differ from that of the skilled readers. The time-gap in RT1 may relate to the general motor (in reading and non-reading related tasks) slowness of response that has been noted in developmental dyslexia [11], [43] perhaps reflecting a deficit in executive functions in dyslexic readers [64]. One cannot rule out, however, that the dyslexics' slowness in RT1 may reflect a deficit in non-motor processing, for example in perceiving the visual task initiation cue [65].

A recently proposed model [66], [67] suggests that there are two distinct cortex-subcortex-cortex loops involved in procedural, motor task, learning: the cortex-striatum-cortex loop is involved in learning to perform task-related movement sequences, and the cortex-cerebellum-cortex loop is involved when basic sensorimotor routines need to be adapted to changing task conditions and task environments (motor adaptation) [67], [68]. Given this model, the current study's data on ET suggest that the basal ganglia loop is largely intact (in function) in adult dyslexic readers. The latest version of the Nicolson and Fawcett hypothesis [3], which directly refers to the Doyon model of skill learning, suggested that dyslexic readers suffer from a cerebellar dysfunction but not from a deficit in the functioning of the basal ganglia. Our results are compatible with this notion. They are not compatible, however, with Nicholson and Fawcett's findings that suggested that manual motor skill learning is deficient in developmental dyslexia [11]. There is an ongoing debate whether the dyslexic reader suffers from an impaired, general domain, procedural learning deficit. Several studies found evidence for a motor deficit among dyslexic readers [69]–[71] while others found no between-group differences [72], [73]. Some authors have suggested that the inconsistent results in studies on sequence learning in dyslexia may be due to factors such as differences in participants' age, the heterogeneity of the subject groups, and comorbidity with other learning disabilities such as ADHD [8], [49], [50]. Participants with clinically relevant symptoms of ADHD were excluded from our study in order to reduce the dyslexic readers' in-group variability. Our results indicate, based on the similarity between the reading-level groups in both RT1 and ET, no evidence for a general domain, procedural learning deficit among the group of dyslexic readers.

In a recent study [5], dyslexic readers were compared to skilled readers in their ability to learn a movement sequence in the Finger Opposition Sequence (FOS) learning task. The study's protocol included a training session and a test 24 hours later. Overall, the dyslexic readers were slower and less accurate. Moreover, on the second day of the study a subgroup of dyslexic readers showed a reduction in accuracy [5] rather than the expected improvement [17]. The authors [5] proposed that some dyslexic readers may express insufficient memory consolidation for the novel motor sequence. A more recent study [42] suggests somewhat similar results in young adults with ADHD who have never received pharmacological treatment trained in the same (FOS learning) task. Evidence for an effective procedural memory consolidation phase was found, although the time-course of expression of delayed gains was longer and atypical [42]. The current results are in line with the notion that memory consolidation processes in individuals with developmental dyslexia may be atypical in some aspects, or more difficult to mobilize, but the potential for effective procedural memory consolidation seems to be preserved.

One should also note, when comparing the different studies, important differences in the nature of the training experience. For example, in the current study, a clear indication of the accuracy of the performance was provided using a visual feedback cue following each successful performance step during training, while in the FOS protocol no feedback was provided.

### The relationships between attention, motor learning and dyslexia

In the current study, the presence of symptoms of ADHD was an exclusion criteria. Nevertheless, a significant correlation was found between ET in each of the three sessions and the DSM-Attention score for the group of skilled readers. Such correlation between attention resources and motor control has been described [74]. However, there was no correlation between ET or any other measure of TST performance and the DSM-Attention score in the dyslexic readers group. This is in line with previous studies suggesting that motor control and attention do not significantly correlate in dyslexic readers. The absence of significant correlation in the current study cannot be accounted for by differences in accuracy of performances (ET means), which were comparable for both reading level groups across all three sessions (Figure 4). Young adult high performing dyslexic readers may be able to reduce the dependence of motor control on general attention resources by way of compensation.

### Learning in the posture control system

The current results indicate a significant difference in the manner and extent to which posture control mechanisms can be recruited to support the performance of a manual task in young adults with developmental dyslexia compared to skilled (typical) readers. All participants in the current study were instructed to maintain a quiet stance position while performing the volitional touch sequence task. The COP measurements, however, show that there were significant changes in posture control, but only in the skilled readers. Thus, in skilled readers, significant changes occurred in measures of posture control – a decrease in postural sway and in sway variance – both effects compatible with the establishment of a task specific posture control routine. A decrease in the variance of performance parameters as a function of training can indicate the establishment of a stable routine and automaticity [31], [56], [75]–[77].

A major role of the posture control system is to support volitional action [78] by maintaining body position and dynamically modulating muscle activities and joint stiffness [79]. A recent study [80] suggests that posture maintenance movements can be combined with volitional task aspects into a unitary movement representation. The current results extend those of Berret et al. [80] in which the postural changes were necessary for task performance, by demonstrating that postural changes that were not entailed by the volitional task could evolve in parallel to the volitional skill and be well maintained across a week long interval. The results of the dyslexic readers may be taken as an indication that volitional skill acquisition can nevertheless proceed, in adults, without specific, measurable, experience-driven modifications in posture maintenance.

Practice effects were more robustly reflected in the decreasing value of the variance of displacement than in the value of displacement itself. Some postural adjustments occurred only early on in training. The variance in the maximal COPx displacement diminished within the first session and between the first and second sessions (Figure 5b). Also, as the bending forward towards the screen was found to be unnecessary, this movement was quickly reduced to a minimum (Figure 5c). Importantly, these adjustments were retained both from the first to the second session and over the week-long interval between the second and third sessions. Thus, the current data support the notion that the posture and balance control system can retain the acquired task-related settings in the form of long-term memory [16].

The dyslexic readers displayed little or no learning effects in their posture control system (Table 3b, Figure 5). This apparent deficit is in line with the notions proposed by Nicolson, Fawcett and colleagues of a (cerebellar) balance control deficit in dyslexia [2], [12], [81], [82]. In a more recent version of their model [3], Nicolson & Fawcett proposed a developmental deficit in a putative cortex-cerebellum-cortex loop [3], [67], [83], an impairment that may prevent dyslexic individuals from developing effective procedural memory, and suggested that motor sequence learning tasks be included in the diagnostic battery for developmental disabilities. The current results suggest that young adult dyslexic readers are not impaired in their ability to acquire and effectively retain experience-dependent procedural knowledge, specifically, the volitional aspects of a task related movement sequence; adult dyslexic readers are capable of learning as robustly as skilled reading peers. The current results do suggest, however, that dyslexic readers are encumbered by a pervasive slowness in the initiation of volitional performance and may be less efficient in adapting the posture control system to support the acquisition of novel movement sequences. These results support the notion of a non-language-related deficit in developmental dyslexia, one related to the recruitment of motor systems for effective task performance, but do not support the notion of a general motor skill learning impairment or a general automatization deficit.
